# Prevalence of Chronic Headache in Croatia

**DOI:** 10.1155/2013/837613

**Published:** 2013-09-01

**Authors:** Vlasta Vuković-Cvetković, Davor Plavec, Arijana Lovrenčić-Huzjan

**Affiliations:** ^1^Department of Neurology, Reference Center for Headaches of the Ministry of Health of Republic Croatia, University Hospital Sestre Milosrdnice, 10 000 Zagreb, Croatia; ^2^Children's Hospital Srebrnjak, Research Department, 10 000 Zagreb, Croatia

## Abstract

*Background*. Chronic headache describes the presence of headache for >15 days per month on average for >3 months and fulfills the rest of the IHS criteria. The prevalence of chronic headache is within the range of 0.5–7.3% worldwide. The aim of this study was to determine the 1-year prevalence of chronic headache in adult Croatian population. *Methods*. The data were collected from a cross-sectional survey of an adult population (>18 years of age) sample. Randomly selected patients from the general population in four Croatian cities were asked to fulfill a self-completed questionnaire. The prevalence of chronic headache was calculated in the sample representing 3 383 769 Croatian adults. *Results*. The total sample included 1542 responders among which 616 were with headache. The 1-year prevalence of chronic headache was 2.4%, and 0.9% of responders declared having headache 30 days per month. According to these results, 81 192 adult inhabitants in Croatia suffer from chronic headache. *Conclusions*. The prevalence of chronic headache in Croatia is comparable to other countries worldwide. These patients require special attention and should be offered multidisciplinary medical support.

## 1. Introduction

Chronic headache (CH) is defined as headaches occurring for >15 days per month for >3 months, in the absence of medication overuse [1]. CH includes four primary headaches: chronic migraine, chronic tension type headache, new daily persistent headache, and hemicrania continua. Population-based epidemiological studies about headaches especially migraine have been carried out in many countries. Few of these studies report the prevalence of CH. Chronic headaches affect approximately 3-4% (range from 0.5–7.3%) of the adult population in western countries [2–4]. The prevalence of CH in the population of the Asia-Pacific regions is similar, from 1.0 to 3.9% [5]. The most frequent subtype is chronic migraine; the prevalence is in the range of 0.2–5, 1% [6, 7]. None of the studies have published data on the incidence of chronic headaches. 

In our previous publication, we have shown that the 1-year age- and sex-adjusted prevalence of migraine in Croatian population was 6.22% (women 8.3%, men 4.0%), of probable migraine 8.78% (women 12.39%, men 5.0%), and of tension type headache 20.65% (women 23.8%, men 17.1%). Age- and sex-adjusted prevalence of combined migraine and probable migraine was therefore 15% (women 20.6%, men 9.0%). Total age- and sex-adjusted prevalence of all headaches was 35.65% (women 44.4%, men 26.1%) [8]. Furthermore, we have shown that women were more likely to suffer from all types of headaches compared with men (OR, 2.2; *P* < 0.0001) [8]. Although chronic headache is highly disabling, it remains largely under diagnosed and undertreated. It is recommended that CH should be diagnosed according to the second edition of the International Classification of Headache Disorders (ICHD-2) [1]. In Croatia, updated national guidelines for the treatment of primary headaches have been published in 2012 [9]. The aim of this study was to determine the 1-year prevalence of chronic headache in adult Croatian population. 

## 2. Methods

The design of the study was a cross-sectional survey of an adult population sample using a self-completed questionnaire. The survey was conducted in four Croatian cities: Zagreb, Split, Osijek, and Dubrovnik from March to October 2006. The study population included adults >18 years of age. General practice registers provide a convenient frame for sampling a local population [10]. In Croatia, 96% of the population is registered with GP. The Croatian population consists of 4 437 000 inhabitants [11]. The prevalence of chronic headache was calculated in the sample representing 3 383 769 Croatian adults (age > 18 years). 

The questionnaires were distributed in general practices to contain a mix of urban, suburban, and rural settings and a spread of social class. Randomly selected patients from the GP practice were asked to fill in the questionnaire. The patients were randomly chosen from the GP's database: after making a contact over the phone, the nurse explained the nature of the survey, and if the patient gave consent to participate in the survey, the questionnaire was sent by mail. 

### 2.1. The Questionnaire Consisted of Three Sections

Section 1 consisted of questions regarding demographic data (age, gender, education, marital status, employment, and place of living) and of questions regarding the presence of headache. The patients have been instructed to respond to Section 1 with answer “no” if they had not suffered from a single headache within the past 3 months or had no more than 1 mild headache attack not requiring treatment. If the respondents answered “yes” to Section 1, they were asked to complete the rest of the questionnaire (Sections 2 and 3). Section 2 included questions which were designed to define the nature of the headache according to the ICHD-2 criteria [1]. Section 3 consisted of questions aiming to assess patterns of headache treatment (not included in this paper). 

The questionnaire was designed using a combination of the literature sources of similar studies, the ICHD-2 criteria, and advice from epidemiologic researchers. The questionnaire has been tested in a pilot study in a general population sample and then distributed to the study population. On return, the questionnaires were checked for the completeness, and questionnaires containing more than 1 unanswered question from either of the three sections were excluded from the final analysis. The diagnosis CH was assessed by two authors (V. V. Cvetković and A. lovrenčić-huzjan), and the 1-year prevalence was calculated. The prevalence of medication overuse headaches (MOH) was not assessed in this study. 

### 2.2. Statistical Analysis

Data analysis was performed using the STATISTICA for Windows release 6.0. Continuous variables were summarized as mean and standard deviation (SD). Categorical variables were summarized as a number (%). In the statistical analysis, the chi-square test was used to compare distribution of categorical variables between subgroups and Student's *t*-test to compare continuous variables. Univariate and multivariate logistic regression analysis was used to identify the odds (OR with 95% CIs) for having a headache, according to the clinical characteristics of the population in question. 

## 3. Results 

The starting sample included 2000 inhabitants from which 1542 questionnaires were suitable for analysis (77.1%). Of the total 1542 responders, 640 (41.5%) filled in the questionnaire indicating that they suffer from headache. Of these, 616 (39.9% of total responders) of questionnaires were suitable for final analysis (consisted of all data regarding headache characteristics). The 1-year prevalence of chronic headache in this study was 2.4% (*n* = 37; 6 men and 31 women). Among patients with recurrent headaches, 6.0% suffer from CH. Even 0.9% of responders declared having headache 30 days per month. According to these results, 81 192 adult inhabitants in Croatia suffer from chronic headache and 30 454 from headaches that occur for 30 days per month. Demographic characteristics of patients with and without headache are shown in Table 1. Mean age of respondents in both groups was 42 (SD, 17) years and for participants with CDH 38 (SD, 14) years. Majority of the respondents had high school education, were married, employed, and lived in a city.

Women were more likely to suffer from CH (OR, 2.82; 95% CI, 1.35–5.90; *P* = 0.0060). Level of education in our study was not significantly different in respondents with or without headache (*P* = 0.675) or with CH (*P* > 0.05). 

Married (45.3%) and divorced (43.9%) respondents were more likely to suffer from headaches than single (36.5%) or widowed (35.7%) (*χ* ^2^ = 13.34, *P* = 0.0095; Table 1), but no significant difference was found for CH (*χ* ^2^ = 1.079, *P* = 0.3006). 

Employed participants were more likely to suffer from headaches (45.6%) than unemployed (39.4%), students (36.7%), or retired (28.6%; *χ* ^2^ = 22.16, *P* < 0.0001; Table 1). 

Students (3.78%) and employed (3.02%) participants were more likely to suffer from CH than unemployed (0.88%) or retired (0.49%; *χ* ^2^ = 8.198, *P* = 0.0421). Place of living (city, suburban, or rural area) was not associated with the prevalence of headaches (*χ* ^2^ = 6.723, *P* = 0.1024; Table 1) or CDH (*χ* ^2^ = 4.038, *P* = 0.2574). Regarding medication usage, participants with CH used 2 drugs per attack for their headache with 28 tablets per month. 

## 4. Discussion

CH is a group of headache disorders whose common features are high frequency of headache and often considerable disability. Criteria for CH warrant having headaches for >15 days per month for >3 months, in the absence of medication overuse. This study revealed that the prevalence of CH in Croatia of 2.4% is comparable to other countries worldwide. CH usually develops as a complication of episodic headaches (mostly migraine or tension type headache) after a period of increasing frequency over months or years. CH is associated with a number of risk factors such as age, family history positive for headache, smoking, obesity, snoring, sleeping problems, head injury, stressful life periods, low educational level, and medication overuse [6, 12–14]. Although the pathophysiology of CH is still poorly understood, research studies have suggested that each of various subtypes of CH may have a different pathogenesis. Several factors are likely to influence headache progression: genetic predisposition, persistent (or recurring) noxious stimuli, trigeminal or cervical pathology, dysfunctional descending modulation of nociception, and repetitive headache attacks that may lead to persistent central sensitization which is directly related to age, duration of illness and migraine attack frequency [15–17]. Some studies implicate that CH is a biobehavioral disorder [18]. 

Chronic migraine is severely disabling and difficult to manage, as affected patients have a substantial number of headaches per month, more comorbid diseases, and more pain and affective disorders than do those with episodic headaches [12, 13, 19]. Diagnosing a chronic headache requires usually three steps, firstly to exclude a secondary headache disorder, secondly to exactly diagnose a specific primary headache (one or more), and thirdly to achieve data on comorbid diseases and medication history. Around 40% of patients attending a specialized headache clinic meet CH criteria, 80% being women; about 60% suffer from chronic migraine, 20% from chronic TTH, and 20% from new daily persistent headache [20]. Among patients with recurrent headaches in this study, even 6.0% suffers from CH. 

Chronic headache imposes substantial burden not only on individual sufferer, but also upon the whole society [21–23]. Studies have shown that CH was consistently associated with greater disability and productivity loss, more consultations, more or longer hospitalizations, and higher direct costs than episodic headaches [23]. Patients with chronic headaches were less likely to be employed full-time, almost twice as likely to be occupationally disabled and twice as likely to have anxiety, chronic pain, and depression [19]. Furthermore, these patients had higher cardiovascular and respiratory risk and were more likely to have heart disease and a history of stroke. Chronic migraine is more likely to develop earlier in life, among females, in midlife, and in households with the lowest annual income [19]. The results of our study have shown that women were almost three times more likely to suffer from CH and that the level of education was not significantly different in respondents with or without headache or for those with CH. However, students and employed participants were more likely to suffer from CH than unemployed or retired. Married and divorced respondents were more likely to suffer from headaches than single or widowed, but no significant difference was found for CH. 

Unfortunately, data on the treatment of chronic headaches are scarce because most prevention trials exclude patients with chronic headaches. However, several evidence-based trials have shown that topiramate and botulinum toxin are efficient in the treatment of chronic headaches, and expert opinion suggests that conventional prophylactic therapy used for migraine and tension type headache may be recommended [24–27]. In our study on the treatment of primary headaches in Croatia, the results have shown that less than half of patients were taking triptans, majority were taking simple analgesics and NSARs, and only 14% was ever taking any kind of prophylactic therapy [28]. The results of this study have shown that participants with CH used 2 drugs per attack for their headache with 28 tablets per month. Although we have not assessed the exact prevalence of MOH in this study, it is probable that a substantial number of our respondents satisfy the criteria for MOH. When looking from the point of a headache sufferer, most headaches require taking a medication, so this easily leads to the development of MOH. Therefore, the majority of patients with CH should probably be given also the diagnosis of MOH and should be treated with more caution. 

Limitations of this study might be the possibility that some individuals classified as CH in the current study would not meet criteria if assessed clinically since the headache groups were defined using a self-completed questionnaire that was based on ICHD-2 criteria and not by clinical assessment; therefore, classification errors are possible. Furthermore, we have not made subclassifications of chronic migraine or chronic TTH because the purpose of this study was to evaluate the chronicity of headaches in the Croatian population. Secondly, this study was performed cross-sectionally, not longitudinally; different types of headaches occurring in the same patient cannot be evaluated cross-sectionally and require longitudinal studies using headache diaries [29]. And thirdly, the prevalence of medication overuse headaches (MOH) was not assessed in this study. 

## 5. Conclusions

The prevalence of chronic headache in Croatia is comparable to other countries worldwide. The relatively high prevalence of CH should be considered a major public health problem. These patients require special attention and should be offered multidisciplinary medical support. Unfortunately, CH is often under recognized, and therefore further extensive epidemiological, pathophysiological, and treatment trails are warranted. 

## Figures and Tables

**Table 1:** Demographic characteristics of patients with and without headache.

Demographic characteristic	Total *n* = 1542 (%)	Headache *n* = 640 (%)	No headache *n* = 902 (%)	*P* value
City				<0.0001
Zagreb	578	245 (38.3)	333 (36.9)	
Split	485	200 (31.3)	285 (31.6)	
Dubrovnik	307	157 (24.5)	150 (16.6)	
Osijek	95	38 (5.9)	57 (6.3)	
Women	926 (60.1)	452 (70.6)	474 (52.5)	<0.0001
Age, mean (SD), yrs	42 (17)	42 (14)	42 (17)	0.4533
Education				0.6754
Elementary	183 (11.9)	78 (12.2)	105 (11.6)	
High school	827 (53.6)	350 (54.7)	477 (52.9)	
College	133 (8.6)	49 (7.7)	84 (9.3)	
University	354 (23)	150 (23.4)	204 (22.6)	
NA	43 (2.8)			
Marital status				0.0095
Married	814 (52.8)	369 (57.7)	445 (49.3)	
Not married	553 (35.9)	202 (31.6)	351 (38.9)	
Widower	56 (3.6)	20 (3.1)	36 (4.0)	
Divorced	66 (4.3)	29 (4.5)	37 (4.1)	
NA	52 (3.4)			
Employment				<0.0001
Employed	895 (58)	408 (63.8)	487 (54.0)	
Not employed	228 (14.8)	90 (14.1)	138 (15.3)	
Student	185 (12)	68 (10.6)	117 (13.0)	
Retired	203 (13.2)	58 (9.1)	145 (16.1)	
NA	31 (2.0)			
Place of living				0.1024
City	987 (64)	402 (62.8)	585 (64.9)	
Suburb	414 (26.8)	189 (29.5)	225 (24.9)	
Rural area	120 (7.7)	43 (6.7)	76 (8.4)	
NA	21 (1.4)			

*NA: not answered.
